# 3-D Super-Resolution Ultrasound Imaging With a 2-D Sparse Array

**DOI:** 10.1109/TUFFC.2019.2943646

**Published:** 2019-09-25

**Authors:** Sevan Harput, Kirsten Christensen-Jeffries, Alessandro Ramalli, Jemma Brown, Jiaqi Zhu, Ge Zhang, Chee Hau Leow, Matthieu Toulemonde, Enrico Boni, Piero Tortoli, Robert J. Eckersley, Chris Dunsby, Meng-Xing Tang

**Affiliations:** ULIS Group, Department of Bioengineering, Imperial College London, London SW7 2AZ, U.K., and also with the Division of Electrical and Electronic Engineering, London South Bank University, London SE1 0AA, U.K; Biomedical Engineering Department, Division of Imaging Sciences, King’s College London, London SE1 7EH, U.K; Department of Information Engineering, University of Florence, 50139 Florence, Italy, and also with the Laboratory of Cardiovascular Imaging and Dynamics, Department of Cardiovascular Sciences, KU Leuven, 3000 Leuven, Belgium; Biomedical Engineering Department, Division of Imaging Sciences, King’s College London, London SE1 7EH, U.K; ULIS Group, Department of Bioengineering, Imperial College London, London SW7 2AZ, U.K; ULIS Group, Department of Bioengineering, Imperial College London, London SW7 2AZ, U.K; ULIS Group, Department of Bioengineering, Imperial College London, London SW7 2AZ, U.K; ULIS Group, Department of Bioengineering, Imperial College London, London SW7 2AZ, U.K; Department of Information Engineering, University of Florence, 50139 Florence, Italy; Department of Information Engineering, University of Florence, 50139 Florence, Italy; Biomedical Engineering Department, Division of Imaging Sciences, King’s College London, London SE1 7EH, U.K; Department of Physics and the Centre for Pathology, Imperial College London, London SW7 2AZ, U.K; ULIS Group, Department of Bioengineering, Imperial College London, London SW7 2AZ, U.K

**Keywords:** 3-D ultrasound imaging, high-frame rate imaging, microbubbles, super-resolution

## Abstract

High-frame-rate 3-D ultrasound imaging technology combined with super-resolution processing method can visualize 3-D microvascular structures by overcoming the diffraction-limited resolution in every spatial direction. However, 3-D super-resolution ultrasound imaging using a full 2-D array requires a system with a large number of independent channels, the design of which might be impractical due to the high cost, complexity, and volume of data produced. In this study, a 2-D sparse array was designed and fabricated with 512 elements chosen from a density-tapered 2-D spiral layout. High-frame-rate volumetric imaging was performed using two synchronized ULA-OP 256 research scanners. Volumetric images were constructed by coherently compounding nine-angle plane waves acquired at a pulse repetition frequency of 4500 Hz. Localization-based 3D super-resolution images of two touching subwavelength tubes were generated from 6000 volumes acquired in 12 s. Finally, this work demonstrates the feasibility of 3-D super resolution imaging and super-resolved velocity mapping using a customized 2-D sparse array transducer.

## I Introduction

VISUALIZATION of the microvasculature beyond the diffraction-limited resolution has been achieved by localizing spatially isolated microbubbles through multiple frames. In the absence of tissue and probe motion, localization precision determines the maximum achievable resolution, which can be on the order of several micrometers at clinical ultrasound frequencies [1], [2]. If motion is present and subsequently corrected postacquisition, then the motion correction accuracy can limit the achievable spatial resolution [3]. Researchers demonstrated the use of 2-D super-resolution ultrasound (SR-US) imaging in many different controlled experiments and preclinical studies using microbubbles [4]–[11] and nanodroplets [12]–[15]. These studies generated super-resolved images of 3-D structures using 1-D ultrasound arrays where super-resolution cannot be achieved in the elevational direction. In addition to this, out-of-plane motion cannot be compensated for, when the data are only acquired in 2-D. However, with the implementation of 3-D SR-US imaging using a 2-D array, diffraction-limited resolution can be overcome in every direction and there is then the potential for 3-D motion tracking and correction.

Many studies have contributed to the development of SR-US imaging methods by improving the localization precision [16], reducing the acquisition time [6], [17], [18], increasing microbubble tracking accuracy [5], [9], [19], and extending the super-resolution into the third dimension [20]–[26]. These developments are explained in detail by a recent review [27]. Researchers mainly employed two different approaches to generate a super-resolution image of a volume by mechanically scanning the volume with a linear probe and stacking 2-D SR-US images or by using arrays that can acquire volumetric information electronically. Errico *et al*. [22] have taken steps toward 3-D with a coronal scan of an entire rat brain by using 128 elements of a custom-built linear array at a frequency of 15 MHz. Motion of the probe was controlled with a microstep motor to generate the 2-D super-resolution images over different imaging planes at a frame rate of 500 Hz. Lin *et al*. [23] performed a 3-D mechanical scan of a rat fibrosarcoma (FSA) tumor using a linear array mounted on a motorized precision motion stage synchronized with the imaging system. They generated the 3-D super-resolution images by calculating the maximum intensity projection (MIP) from all 2-D super-resolution slices, acquired using plane-wave imaging with a frame rate of 500 Hz. Zhu *et al*. [25] used a similar approach with Lin *et al*. [23] to scan a rabbit lymph node using a high-precision motorized translation stage with an 18-MHz linear array at a frame rate of 500 Hz. They generated a 2-D MIP of the whole lymph node with super-resolution and super-resolved velocity mapping. Although subdiffraction imaging has not been published using a 2-D imaging probe with a high volumetric imaging rate, 3-D super-resolution has been achieved by previous studies. O’Reilly and Hynynen [20] used a subset of 128 elements from a 1372-element hemispherical transcranial therapy array at a rate of 10 Hz. They generated the 3-D super-resolution images of a spiral tube phantom through an *ex vivo* human skullcap at an imaging center frequency of 612 kHz. Desailly *et al*. [21] implemented a plane-wave ultrafast imaging method using an ultrasound clinical scanner with 128 fully programmable emission-reception channels. They placed two parallel series of 64 transducers to image microfluidic channels and obtained 3-D superlocalization by fitting parallel parabolas in the elevation direction. Christensen-Jeffries *et al*. [24] generated volumetric 3-D super-resolution at the overlapping imaging region of two orthogonal transducers at the focus. They used two identical linear arrays to image subdiffraction cellulose tubes using amplitude-modulated plane-wave transmission at 3 MHz with a frame rate of 400 Hz. Heiles *et al*. [26] performed 3-D ultrasound localization microscopy on a wall-less bifurcation phantom with 200- and 400-*μ*m channels and calculated 3-D microbubble trajectories. They used a 1024-element matrix array probe connected to four ultrasound systems with 256 transmit and 128 multiplexed receive channels to image the phantom at 9 MHz with a volume rate of 500 Hz.

The development of high-speed programmable ultrasound systems and 2-D arrays created new opportunities for volumetric imaging with high spatiotemporal resolution. In parallel to these hardware developments, novel 3-D imaging methods based on small numbers of transmit–receive pairs enabled a more reliable visualization of tissue volumes [28], the analysis of fast and complex blood flow in 3-D [29]–[32], the characterization of mechanical properties of tissue by 4-D shear-wave imaging [28], [33], the tracking of the pulse wave propagation along the arterial wall [34], the estimation of 4-D tissue motion [35], and other *in vivo* transient events. These technological advances in 3-D imaging also offer new opportunities for SR-US. Although volumetric imaging methods have already shown significant benefits for various ultrasound imaging applications, 3-D imaging with large 2-D arrays requires a high number of hardware channels and huge computational power.

In this study, we demonstrate the feasibility of 3-D super-resolution imaging and super-resolved flow velocity mapping using a density-tapered sparse array instead of a full 2-D array to reduce the number of channels and hence the amount of data while maintaining the volumetric imaging rate. A similar approach was in previous nonsuper-resolution studies on minimally redundant 2-D arrays [36] and sparse 2-D arrays [37]–[41], but it uses a greater number of elements to improve transmit power and receive sensitivity. Our method significantly differs from row–column addressing and multiplexing approaches since it maintains simultaneous access to all probe elements through independent channels. The sparse array was designed specifically for high volumetric rate 3-D super-resolution ultrasound imaging based on a density-tapered spiral layout [42], [43]. The capability of the 2-D sparse array for 3-D SR-US imaging was demonstrated in simulations and experiments.

## II Materials and Methods

### A 2-D Sparse Array

**Fig. 1:**
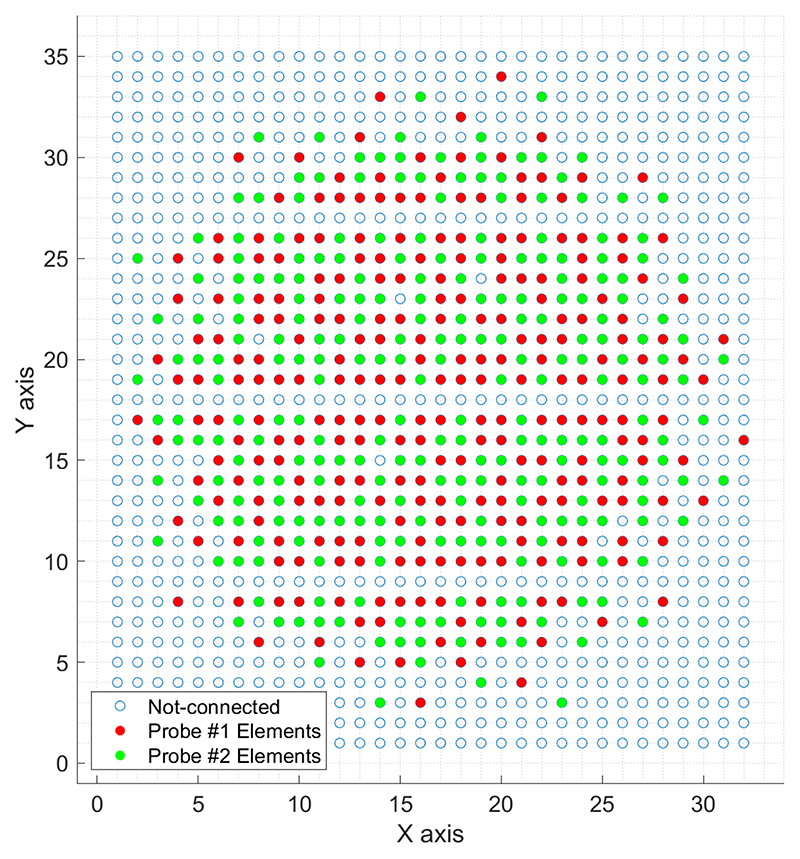
Layout of the 2-D sparse array with red and green circles showing the chosen elements. The pitch between the consecutive elements in the *x-* and *y*-directions is 300 *μ*m. Inactive rows (9, 18, and 27) are due to the manufacturing limitations and are not related to the density-tapered 2-D spiral method.

A 2-D sparse array was designed by selecting 512 elements from a 32 × 35 gridded layout of a 2-D matrix array (Vermon S.A., Tours, France), as shown in Fig. 1. It was fabricated with an individual element size of 300 × 300 *μ*m, a center frequency of 3.7 MHz, and a bandwidth of 60%. In the *y*-direction, row numbers 9, 18, and 27 were intentionally left blank for wiring, and hence, the total number of available elements is 1024. The method to select the location of sparse array elements is based on the density-tapered 2-D spiral layout [42]. This method arranges the elements according to the seeds generated from Fermat’s spiral function with an additional spatial density modulation to reduce the sidelobes of the transmitted beam profile. This deterministic, aperiodic, and balanced positioning procedure guarantees uniform performance over a wide range of imaging angles.

It is not possible to connect all 512 elements to a single ultrasound probe adapter. Therefore, two sparse array layouts, hereinafter referred to as Aperture#1 and Aperture#2, were designed as shown with red and green elements in Fig. 1. Both sparse arrays were based on an ungridded, 10.4-mm-wide spiral with 256 seeds [42], whose density tapering was modulated according to a 50%-Tukey window. The elements belonging to Aperture#1 were selected among those of the Vermon 2-D matrix array, by activating the available elements whose positions were closest to the ideal positions of the ungridded spiral. Similarly, the elements belonging to Aperture#2 were also selected among those of the Vermon matrix array, but excluding those that were already assigned to Aperture#1. The two layouts were connected to two independent connectors (model DLP 408, ITT Cannon, Irvine, CA, USA) so that an approximation of a 256-element density-tapered spiral array could be driven by an independent ULA-OP 256 system [44], [45]. Moreover, by synchronizing two ULA-OP 256 systems to simultaneously control the two layouts, a 512-element dense array (Aperture#1 + Aperture#2) with integrated Tukey apodization could be driven.

### B Experimental Setup

Two ULA-OP 256 [44], [45] systems were synchronized to transmit nine plane waves from the 512 selected elements. Plane waves were steered within a range of ±10° with a step size of 10° in both the lateral and elevational directions. A three-cycle Gaussian pulse with a 3.7-MHz center frequency was used for imaging. Prebeamforming raw data for nine angles were acquired with a pulse repetition frequency (PRF) of 4500 Hz. These nine volumetric acquisitions were coherently compounded to construct imaging volumes at a frame rate of 500 Hz. This frame rate was high enough to limit intravolume motion artifacts due to moving microbubbles in flow [46]. For the experiments with slow flow rate, a total of 12 000 volumetric ultrasound frames were acquired in 24 s at an mechanical index (MI) of 0.055. For the experiments with fast flow rate, a total of 6000 volumetric ultrasound frames were acquired in 12 s at an MI of 0.055.

**Fig. 2:**
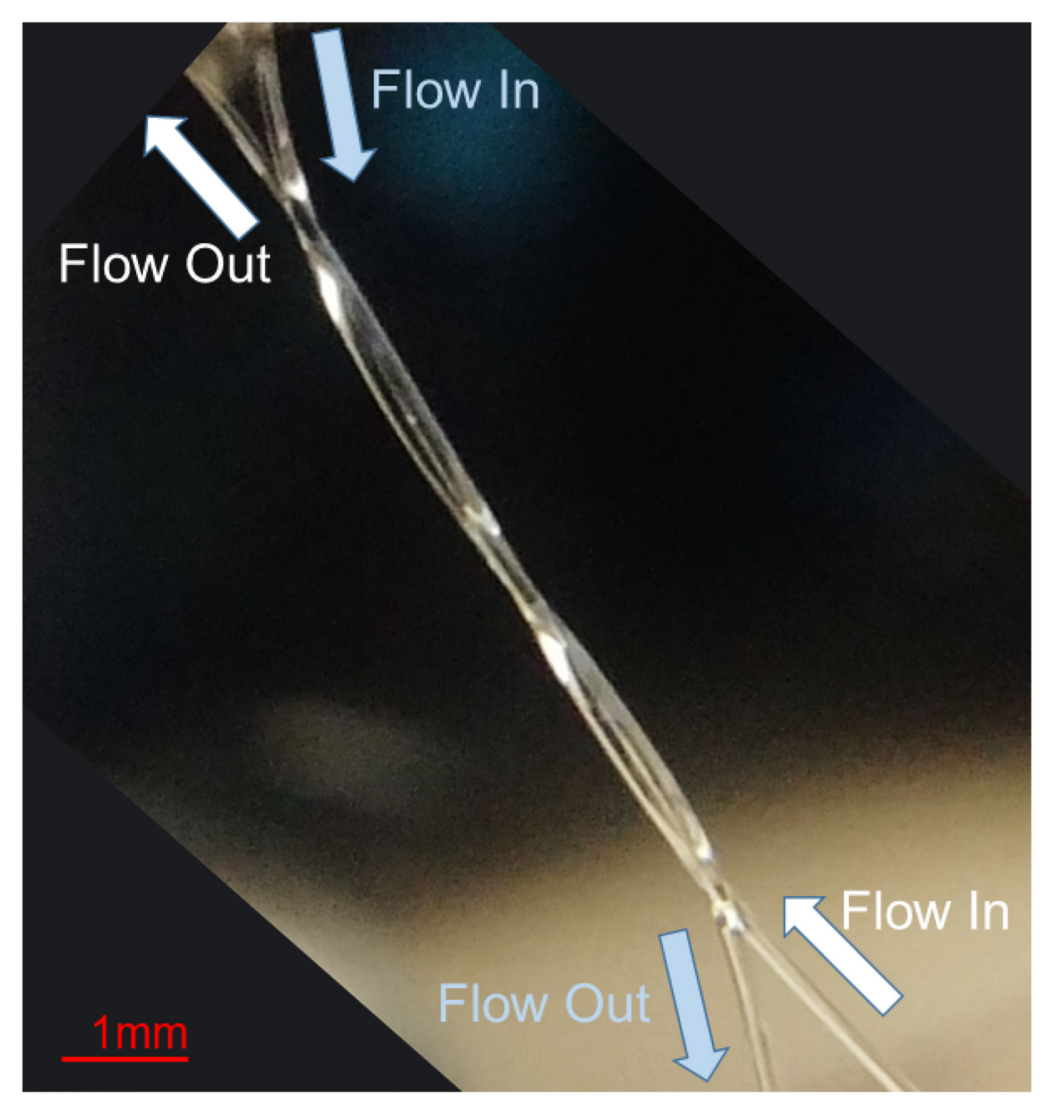
Optical image of two 200-*μ*m cellulose tubes arranged in a double helix pattern. To create this pattern, two tubes were wrapped around each other which created contact points that are visible in the optical image. Both tubes had constant microbubble flow in the opposite directions.

The microvessel phantom was made of two 200 ± 15 *μ*m Hemophan cellulose tubes (Membrana, 3M, Wuppertal, Germany) with a wall thickness of 8 ± 1 *μ*m. Two tubes were arranged in a double helix shape at a depth of 25 mm, as shown in Fig. 2. The volumetric B-mode imaging was performed without microbubble flow inside these tubes. For SR-US imaging, Sonovue (Bracco S.p.A, Milan, Italy) solution was flowed through both tubes in the opposite directions using a dual-infusion pump in the withdrawal mode with a constant flow rate that produced a mean microbubble velocity of 11 or 44 mm/s, where the maximum microbubble velocity is expected to be 22 or 88 mm/s inside the tubes with laminar flow. The concentration of the microbubble solution was initially set to 1:500 (Native microbubble solution: Water) and gradually diluted until reaching a suitable concentration for SR-US imaging at 1:2000.

### C Super-Resolution Processing and Velocity Calculations

The RF signals obtained by each aperture (#1 and #2) were separately beamformed. First, singular value decomposition was performed on these data sets to separate the microbubble signal and the echoes from the tube [47]. After isolating the microbubble signals, data acquired from two probes were combined offline using the acoustic sub-aperture processing (ASAP) method [48]. By processing and beamforming the data from two apertures separately with the ASAP method, an SNR improvement (2.9–5.1 dB) was achieved, since a noisy signal resembling a microbubble echo is unlikely to occur simultaneously on both beamformed volumes from different systems.

After combining the beamformed data from both apertures to reconstruct a single volume, an intensity threshold was applied to further reduce the noise level by removing the data below the threshold value. After thresholding, superlocalization was performed on the remaining data that may represent a microbubble. In addition to detecting their locations, the volume of every microbubble echo above the intensity threshold was calculated. To remove the localizations that may belong to multiple microbubbles, detections were discarded if their volume was two times larger than the volume of the 3-D B-mode point spread function (PSF).

Velocities of the detected microbubbles were traced using the nearest-neighbor method between the consecutive frames. First, the Euclidean distance between the target microbubble from frame *n* and the detected microbubbles from frame *n* + 1 is calculated [49]. This distance value was used to find the nearest-neighbor microbubble in the consecutive frame without any weighting [50]. The Euclidean distance between the paired microbubbles was multiplied with the frame rate to estimate the microbubble velocity. Velocity values of multiple microbubbles corresponding to the same spatial point were averaged. An additional measure was used to filter incorrect pairings. If, in consecutive frames, there was more than 50% deviation in volume size between the microbubble echoes, the velocity track was replaced with the next closest microbubble pair after the same size comparison. To accelerate the tracking, a search window was set to allow a maximum microbubble velocity of 100 mm/s. This velocity value is larger than the velocity profile expected in human microcirculation, where Tuma *et al*. [51] reported a mean velocity of 7–35 mm/s in small arteries with a diameter of 40–130 *μ*m and 5–25 mm/s in small veins with a diameter of 60–180 *μ*m in human eye measured by laser Doppler velocimetry.

## III Results

### A 2-D Sparse Array Simulation Results

**Fig. 3:**
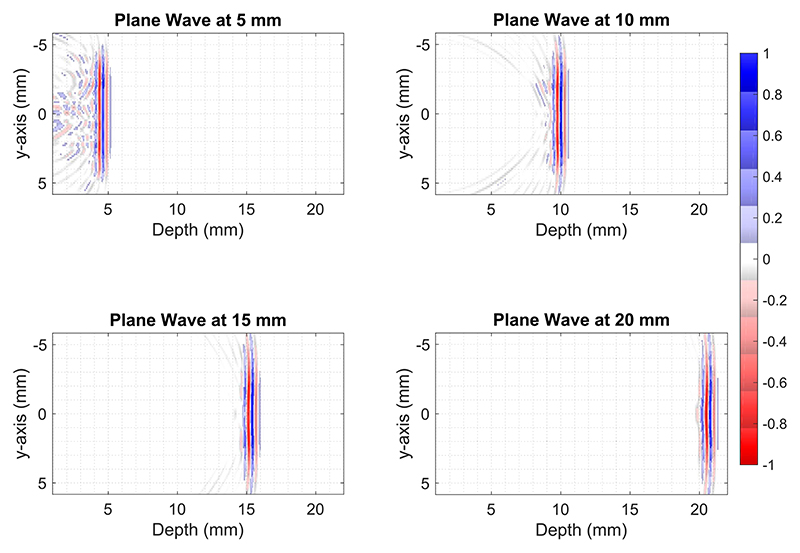
Simulated plane-wave propagation at 5-, 10-, 15-, and 20-mm depths. A three-cycle Gaussian pulse was simultaneously transmitted from 512 elements of the 2-D array. All the panels are normalized to their respective maximum.

**Fig. 4:**
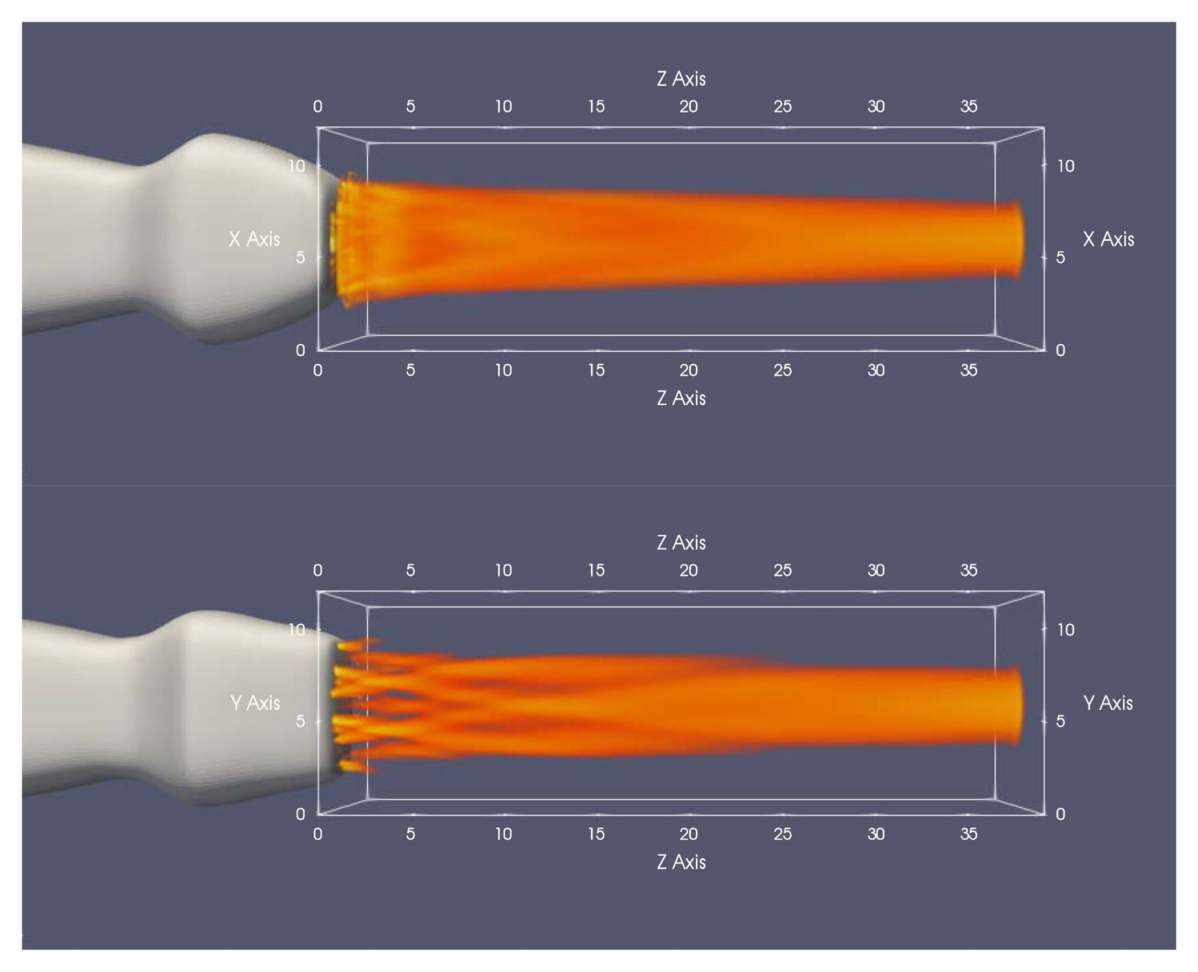
Simulated 3-D ultrasound field radiated from the sparse array is shown from the *xz*-view (top) and the *yz*-view (bottom), where *z*-axis represents the depth.

To evaluate the feasibility of the proposed approach, planewave propagation from the 512-element sparse array was simulated at different depths as shown in Fig. 3 using Field II [52], [53]. The radiated ultrasound field within the first 5-mm depth [see Fig. 3 (top left)] is a combination of a plane wave and a dispersed tail, which is a result of missing rows. At the depth of 10 mm, as shown in Fig. 3 (top right), the tail resembles a superposition of multiple edge waves as a result of discontinuities in the array. At this point, the radiated beam shape is not suitable for generating a good quality image. Around 15-mm depth, as shown in Fig. 3 (bottom left), the tail becomes less prominent and edge waves diminish below −14 dB; however, it can still produce image artifacts, as demonstrated in [54]. Further away from the transducer, the residual waves behind the wavefront disappear and the ultrasound field becomes more uniform, which is suitable for plane-wave imaging after 20-mm depth, as shown in Fig. 3 (bottom right). The 3-D simulations displayed in Fig. 4 also support the same conclusion; due to the choice of elements and three unconnected rows, the ultrasound field is not uniform for the first 20 mm.

### B 3-D Super-Resolution Experimental Results

Before performing the experiments on a cellulose microvasculature phantom, the imaging performance of the 2-D sparse array was characterized with a point target using the tip of a 100-*μ*m metal wire. The full-width-half-maximum (FWHM) of the 3-D B-mode PSF was measured as 793, 772, and 499 *μ*m in the *x*-, *y*-, and *z*-directions, respectively, by using linear interpolation [55]. The localization precision was measured to be the standard deviation of the localization positions over 100 frames. The 3-D superlocalization precision of the overall system at 25 mm was found to be 18 *μ*m in the worst imaging plane (*x*-direction), where the imaging wavelength is 404 *μ*m in water at 25 °C.

**Fig. 5:**
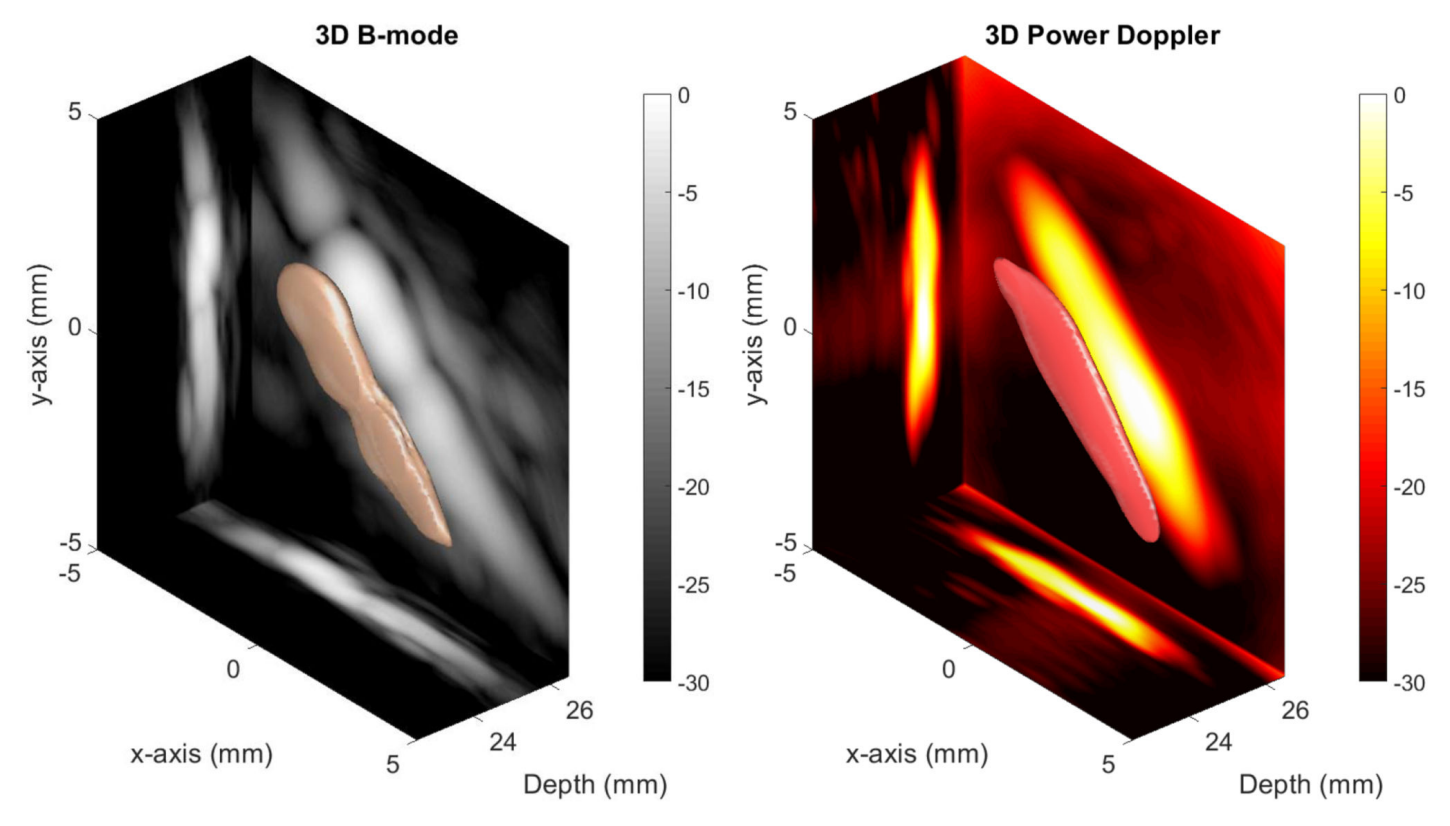
Left: 3-D ultrasound B-mode image is plotted in copper at −10-dB isosurface level. Right: 3-D power Doppler image is plotted in red at −10-dB isosurface level. The 2-D maximum intensity projections with a 30-dB dynamic range are overlaid on the volumetric images.

The volumetric B-mode image of two cellulose tubes without microbubble flow is shown in Fig. 5 (left). In addition to the 3-D visualization of the structure displayed in copper color, 2-D MIP slices in three directions were plotted. After this measurement, microbubbles were flown through the tubes and a 3-D power Doppler image was generated, as shown in Fig. 5 (right) using singular value decomposition [47]. It was not possible to visualize the two separate 200-*μ*m tubes in the 3-D B-mode and power Doppler images.

**Fig. 6:**
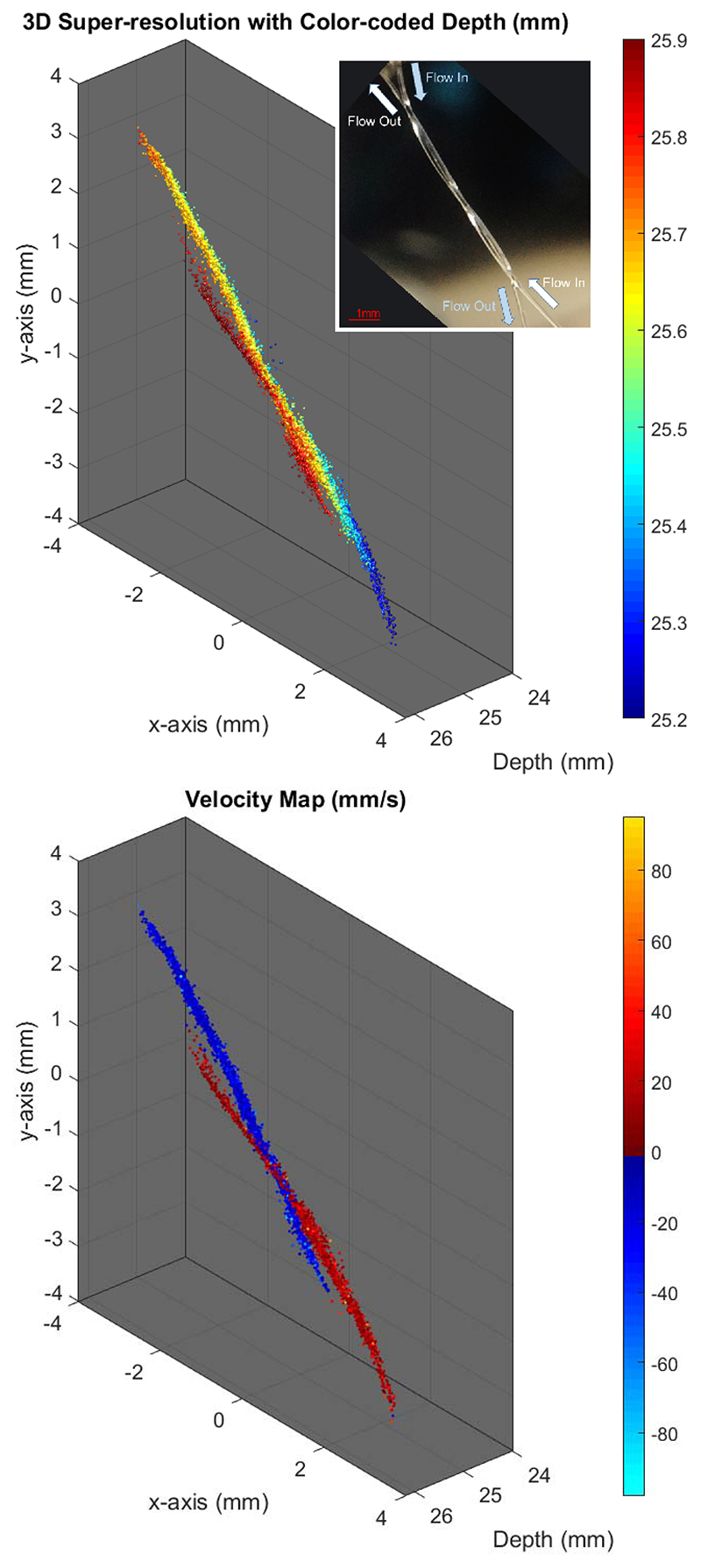
Experimental setup of two 200-*μ*m tubes arranged in a double helix shape with a mean microbubble velocity of 11 mm/s. Top: 3-D super-resolution image was generated with 10626 localized microbubbles from 12000 volumes. Depth-encoded colorscale is added to improve the visualization. Inset: optical image of the setup http://dx.doi.org/10.1109/TUFFC.2019.2943646/mm1. Bottom: velocity maps (positive toward increasing *y*-direction) of tracked microbubbles flown through the tubes. Supplementary material: Fig6(top)_3D_SR_depthencoded_Vmean11mms and Fig6(bottom)_3D_SR_VelocityMap_Vmean11mmshttp://dx.doi.org/10.1109/TUFFC.2019.2943646/mm4.

Figs. 6 (top) and 7 (top) show the 3-D super-resolved volume of the imaged subwavelength structures by combining localizations from all acquired frames. In the experiments with a mean microbubble velocity of 44 mm/s, a total of 9562 microbubbles were localized within the 6000 compounded volumes. For the slow experiments with a mean velocity of 11 mm/s, a total of 10626 microbubbles were localized within the 12 000 compounded volumes. Due to the large number of localizations, the 3-D structure of the tubes cannot be clearly visualized in a single 2-D image. To improve the visualization, 3-D SR-US images are plotted with depth information color-coded in the image.

Figs. 6 (bottom) and 7 (bottom) show the velocity profiles of tracked microbubbles. For the experiment with the mean flow velocity of 11 mm/s, 4641 microbubble pairs out of 10 626 microbubbles were traceable from the consecutive frames using a nearest-neighbor method. For the experiment with the mean flow velocity of 44 mm/s, 3359 microbubble pairs out of 9562 microbubbles were traceable. Using these microbubble tracks, two subwavelength tubes with opposing flows were easily distinguishable by color-coding the direction of their velocity vectors. The 3-D velocity maps are displayed from different viewing angles in the supplementary video for better visualization.

**Fig. 7:**
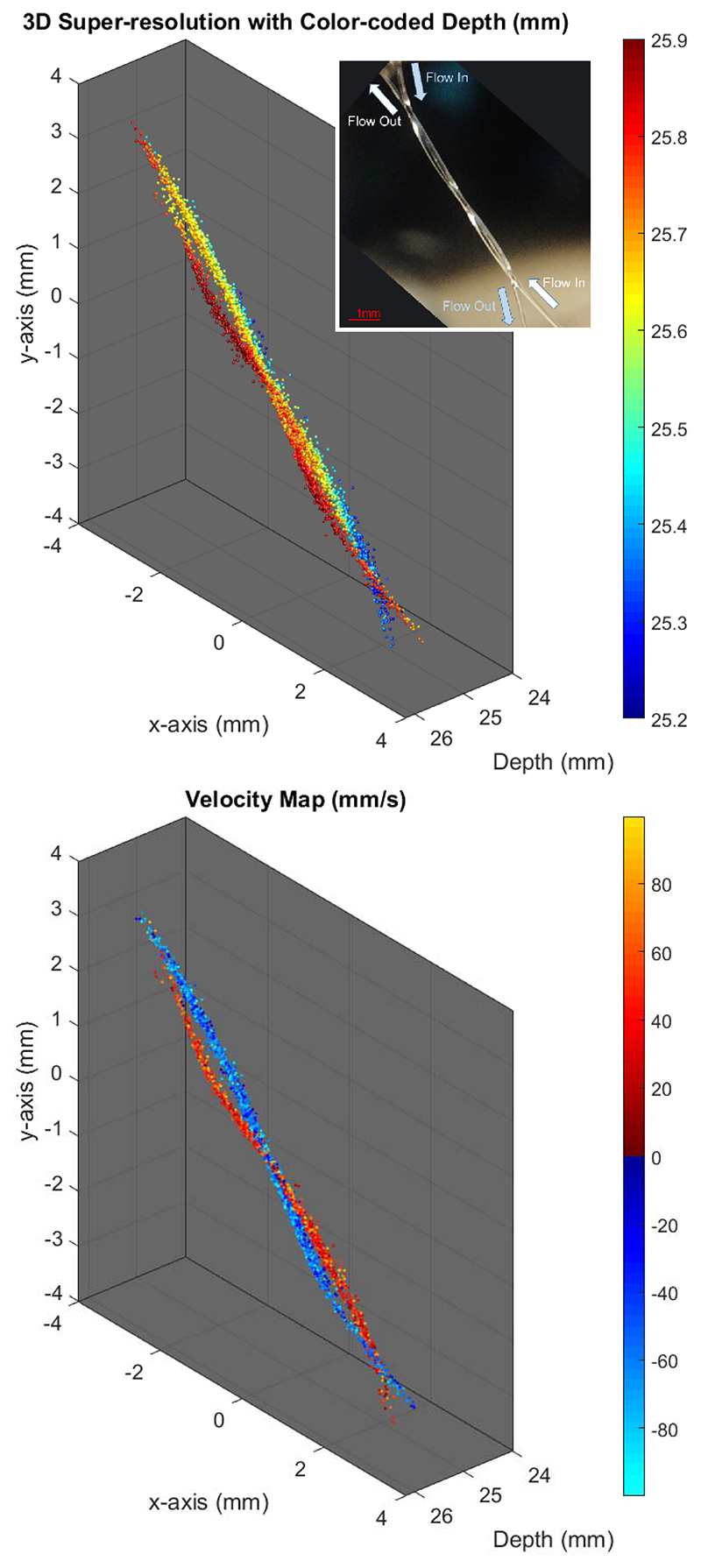
Experimental setup of two 200-*μ*m tubes arranged in a double helix shape with a mean microbubble velocity of 44 mm/s. Top: 3-D super-resolution image was generated with 9562 localized microbubbles from 6000 volumes. Depth-encoded colorscale is added to improve the visualization. Inset: optical image of the setup http://dx.doi.org/10.1109/TUFFC.2019.2943646/mm2. Bottom: velocity maps (positive toward increasing *y*-direction) of tracked microbubbles flown through the tubes. Supplementary material: Fig7(top)_3D_SR_depthencoded_Vmean44mms and Fig7(bottom)_3D_SR_VelocityMap_Vmean44mmshttp://dx.doi.org/10.1109/TUFFC.2019.2943646/mm3.

**Fig. 8:**
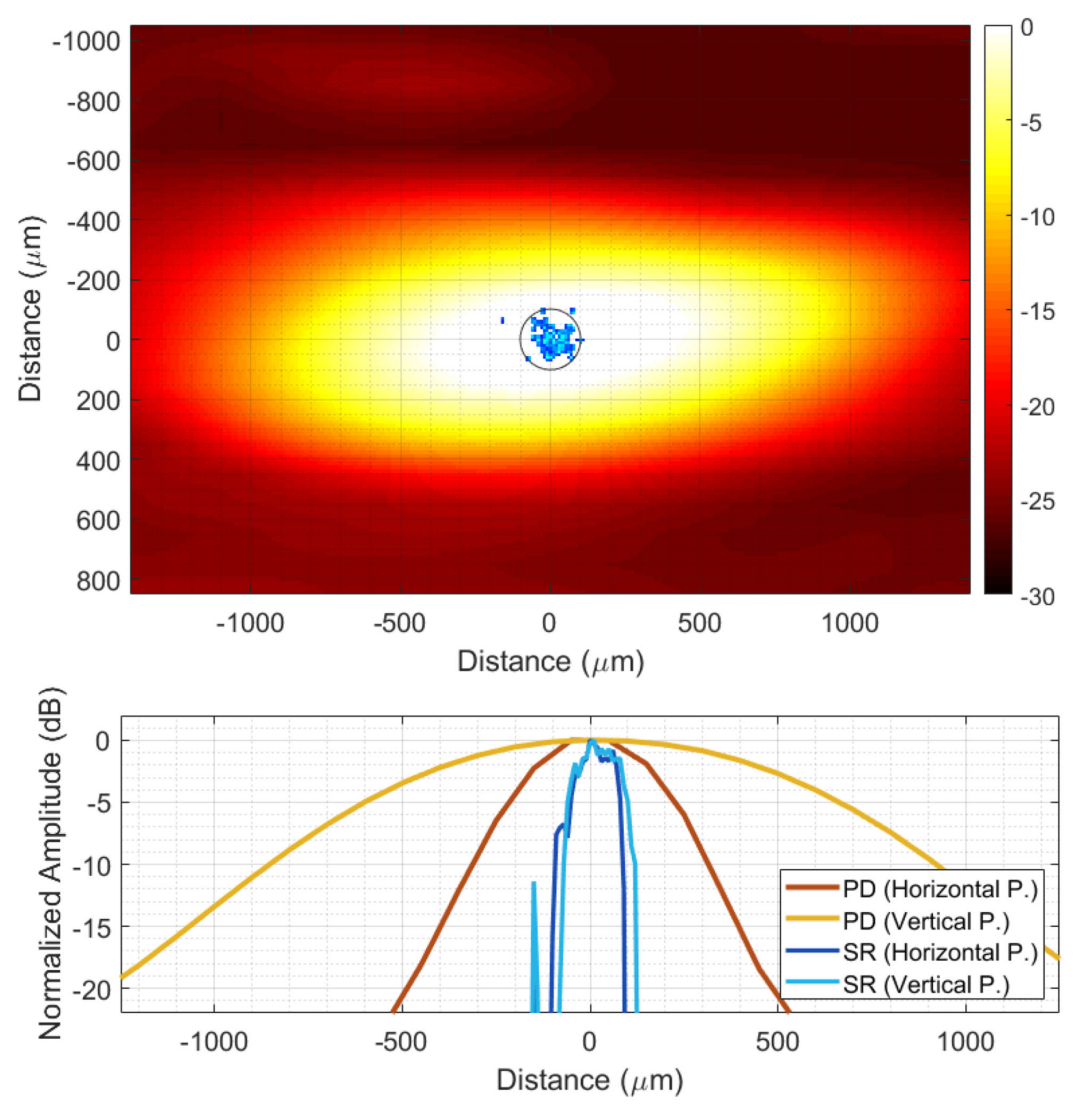
Top: MIP of the power Doppler image belonging to a 0.5-mm-long section of the tube projected into a 2-D plane that is orthogonal to the direction of the flow. The super-resolution image was projected into the same 2-D plane and overlaid on the power Doppler image in blue colors. Black circle represents the 200-*μ*m tube circumference. Bottom: FWHM of the tube is measured as 1381 and 495 *μ*m from 1-D projections in the horizontal and vertical directions of the top plot, respectively. The super-resolution FWHM of the tube is measured as 136 and 165 *μ*m from 1-D projections in the horizontal and vertical directions of the top plot, respectively.

The thickness of the imaged tubes was measured at the inlet where the tube is clearly isolated in the 3-D SR-US image around the coordinates (*x* = 2 mm, *y* = −3 mm). To perform the thickness measurement, a 0.5-mm-long section of the imaged tube was chosen and projected into a 2-D plane that is orthogonal to the direction of the tube, as shown in Fig. 8 (top) both for power Doppler image and 3-D SR-US image from Fig. 7 (top). Fig. 8 (bottom) shows the 1-D MIP in the horizontal and vertical directions where the FWHM of the super-resolved tube was measured as 136 and 165 *μ*m and the −20 dB width of the super-resolved tube was measured as 194 and 204 *μ*m, respectively, for the experiments with a mean microbubble velocity of 44 mm/s. The other experiments with slower flow velocity had similar results with an FWHM measured as 135 and 158 *μ*m in the horizontal and vertical directions from Fig. 6 (top). In the 3-D power Doppler image, two touching tubes appeared as a single scattering object with an FWHM of 1381 and 495 *μ*m in the horizontal and vertical 1-D projections, respectively.

**Fig. 9:**
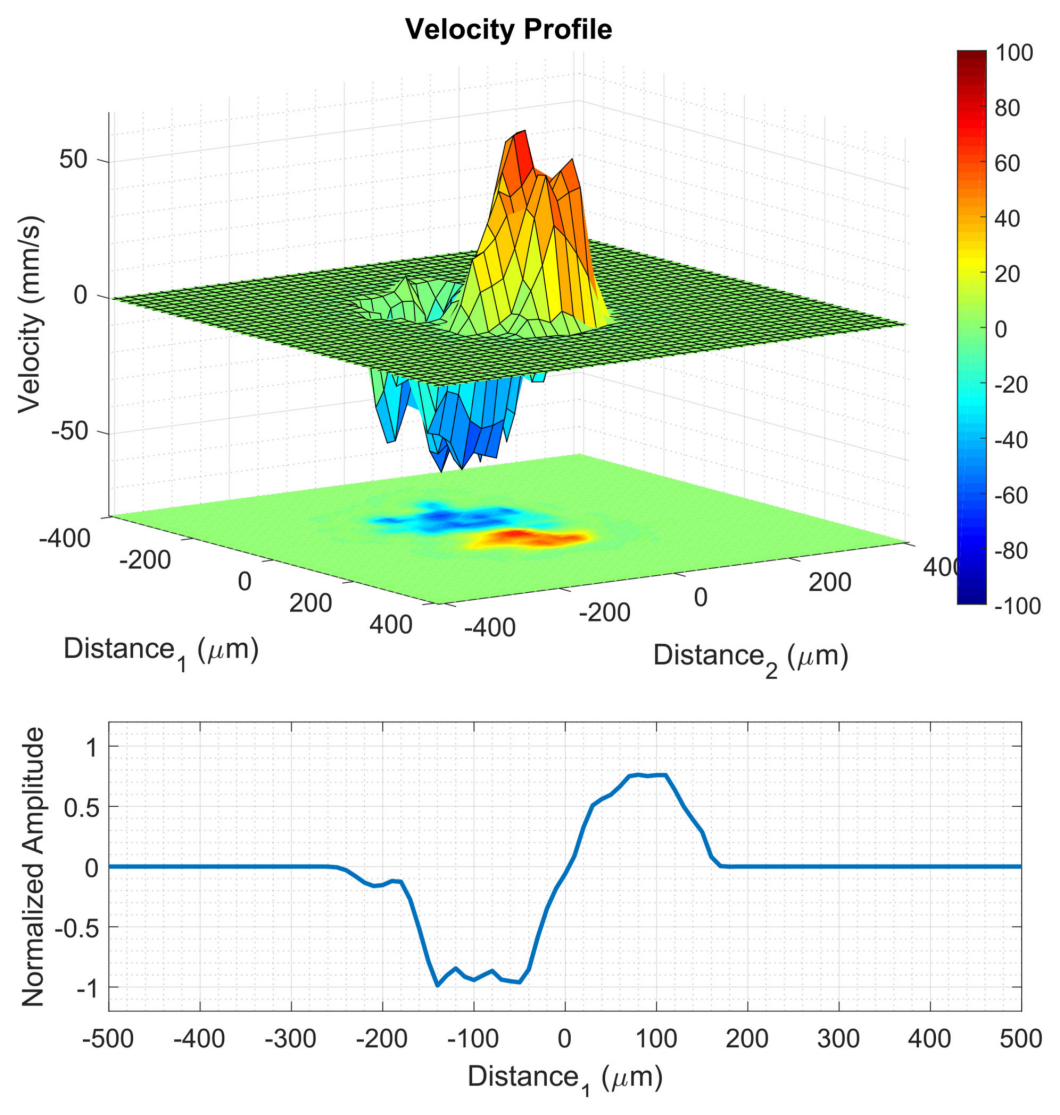
Top: 3-D velocity profiles of microbubbles are plotted as a surface plot from Fig. 7 (bottom) at (*x* = 1 mm, *y* = −1 mm) with a plane orthogonal to both flows. The MIP is plotted below as a 2-D plane. Bottom: 1-D projection toward Distance_1_ shows the separation between the negative and positive flows, where the peak-to-peak distance between the two opposing velocity tracks is 200 *μ*m.

The velocity profiles of microbubbles with two touching tubes were analyzed at different locations over the whole volume, where Fig. 9 shows the velocity profiles at (*x* = 1 mm, *y* = −1 mm) from Fig. 7 (bottom). To perform this analysis, the 3-D volume was sliced with a 2-D plane that is orthogonal to both flows at different locations. In addition to the 2-D plane shown in Fig. 9, the peak-to-peak distance between two opposing tracks was measured at four different locations as 190 ± 30 *μ*m from their 1-D projection, as shown in Fig. 9 (bottom). Microbubble tracking made the separation between the tubes clearer when the tubes are in contact around the central section of the 3-D SR-US and velocity maps shown in Figs. 6 and 7.

## IV Discussion

A better 3-D image quality may be achieved by using a large number of independent array elements with the fastest possible volumetric imaging rate; however, this requires the same number of hardware channels as the number of elements and the ability to process very large stacks of data. Due to high cost, full 2-D array imaging using an ultrasound system to control very large numbers of independent elements has only been used by a few research groups [28], [33], [56], [57]. These systems had 1024 channels capable of driving a 32 × 32 2-D array with at least four connectors. Even some of these systems had one of two transducer elements multiplexed in reception [28], [33]. Many researchers have developed methods to use a large number of active elements with fewer channels (usually between 128 and 256)to reduce the cost and complexity of the ultrasound systems and the probes. It has been demonstrated in several studies that row–column addressed matrix arrays [54], [58]–[60], microbeamformers [61]–[63], and channel multiplexing can be an alternative to fully addressed 2-D matrix arrays. However, these methods have less flexibility and limitations due to the elements not being continuously connected to the ultrasound system.

In this article, a 2-D sparse array imaging probe has been developed for 3-D super-resolution imaging. This has addressed the main limitation of the existing 2-D imaging of poor spatial resolution in the elevational plane. In addition to super-resolution imaging, 3-D velocity mapping was implemented to reveal the flow inside the microstructures. Using the sparse array approach instead of the full matrix array reduced the number of channels to half and, hence, the connection issues, cost, and data size while still achieving the same volumetric acquisition speed since all elements of the 2-D spiral array are always connected to the system. Although this approach can reduce the maximum achievable transmit pressure and receive sensitivity, it is not a significant issue with SR-US due to the low pressure required and the high sensitivity achievable in microbubble imaging. In terms of B-mode image resolution, the axial resolution is comparable, since both arrays have the same bandwidth, while a slightly worse lateral resolution is expected for the sparse array, since the full matrix array has a larger equivalent aperture size. It is hard to distinguish the grating lobes and the sidelobes of a sparse array, but here we consider the unwanted leakage outside the main lobe as grating lobes since it is as a result of element-to-element spacing and as sidelobes since it is as a result of finite aperture size. The highest grating lobe of the full matrix array is predicted to appear at ±8° with an amplitude as high as 17% of the main lobe, calculated using the array factor equation in [64]. A sparse choice of elements spreads the grating lobes to a wider range due to the irregular placement of elements, where the highest grating lobe will appear at ±18° with an amplitude as high as 16% of the main lobe. The sidelobe and edge wave suppression characteristics of the sparse array will outperform an unapodized full matrix array due to the integrated apodization [54], although the fixed apodization might be a limitation for some applications. Both arrays will have higher grating lobes in the *y*-direction due to the three inactive rows.

In this study, 3-D super-resolution images and 3-D superresolved velocity maps have been generated from 12- and 24-s acquisitions with a 3-D ultrasound B-mode imaging rate of 500 Hz. The implemented velocity estimation technique made use of the whole data set to calculate microbubble velocities. When all velocity estimations were combined from multiple frames, Figs. 6 (bottom) and 7 (bottom) revealed the average flow inside the microvessel phantom. The presented 3-D SR-US method can estimate the average blood flow rate, blood vessel diameter, and vascular density (not shown in this study), which might be used to find the structural differences between the normal and tumor microvascular networks and even identify angiogenic vessels [65]. However, the used velocity estimation technique cannot achieve a high temporal resolution to visualize the pulsatile flow. Although flow is not pulsatile in microvessels below a certain size, pulsatile flow can be observed in microvessels around the proximal sections of major organs. Temporal changes of velocity in these microvessels can be clinically important. Low temporal resolution is a common limitation for the existing localizationbased super-resolution imaging methods and researchers are developing new methods to achieve fast super-resolution ultrasound imaging. Bar-Zion *et al*. [6] employed higher order moments to increase image resolution. Their statistical model was used as a postprocessing technique for improving the quality of displayed images and achieving a subsecond frame rate. In a more recent study, the same authors proposed a different method to exploit the sparsity of the underlying vasculature in the correlation domain [18]. The sparse recovery processing method is demonstrated by using the correlation-based images calculated from the low-resolution measurements. Although not demonstrated yet, their method might be useful for finding changes in microvascular velocity profiles due to a temporal resolution of 25 Hz. In a different study, Yu *et al*. proposed a new approach to improve temporal resolution by employing deconvolution and spatiotemporal-interframe-correlation-based data acquisition [66]. They used the number of detected moving microbubbles to predict the cardiac phase, after extracting nonstationary microbubbles with an eigen-based spatiotemporal tissue rejection filter. They assumed that microbubbles are less likely to flow at diastolic phase and microbubbles are faster toward the systole phase. Their method synchronized sequentially acquired multiple data sets to form a single cardiac cycle event with high temporal resolution, where the cardiac pulsation was estimated by the number of detected microbubbles. These are potential methods that may improve the velocity estimation performance and functionality of super-resolution images by achieving high temporal resolution, although further study is required to demonstrate experimentally that such techniques can achieve a similar spatial resolution to those localization-based methods.

**Fig. 10:**
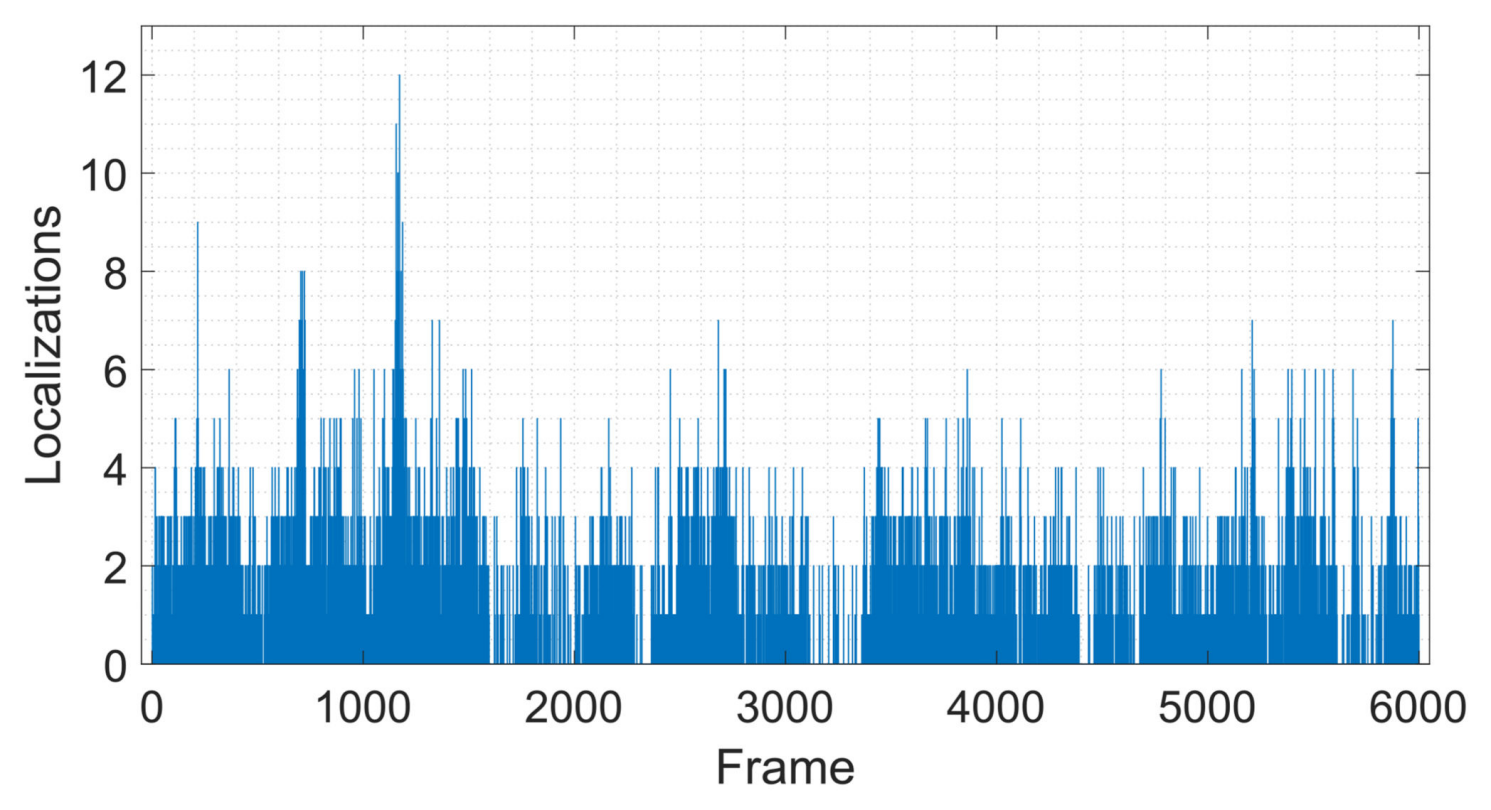
Number of localized microbubbles in each 3-D acquisition frame for the experiments with a mean flow velocity of 44 mm/s. These localizations were used to generate the super-resolution image shown in Fig. 7.

Using the plane-wave imaging method instead of lineby-line scanning increases the temporal resolution of the volumetric imaging. Faster 3-D image acquisition provides a higher microbubble localization rate and improves velocity estimations due to more frequent sampling. Fig. 10 shows the histogram of localized microbubbles in each frame for the results presented in Fig. 7. For a relatively small microvessel phantom of two 200-*μ*m tubes shorter than 10 mm, around 1.6 microbubbles were localized with a precision suitable for subdiffraction imaging at a volumetric imaging rate. At this high insonation rate, even at a relatively low MI of 0.055, many microbubbles were destroyed before reaching the end of the imaging region, which can be seen at the outlet of the tubes in Fig. 6. In this case, a microbubble traveling with a velocity of 11 mm/s through the imaging region (the length of the diagonally aligned tube inside the imaging region was around 10 mm) was exposed to over 4000 ultrasound pulses at a PRF of 4500 Hz. However, for the flow velocity of 44 mm/s, microbubbles were exposed to four times less ultrasound pulses and tube shape is visualized better at the outlets, as shown in Fig. 7. Although the average number of localizations was lower due to potential microbubble disruption, microbubbles were tracked with a higher efficiency at the slower flow rate. The percentage of microbubbles that were followed over two or more volumes with the tracking algorithm used was 70% and 87%, for the experiments with a flow velocity of 44 and 11 mm/s, respectively. Two potential explanations for the higher tracking rate for slower flow are: 1) slower microbubbles relative to the image acquisition speed are easier to track between the successive image volumes and 2) the PSF volume changes when the same microbubble is imaged at different locations, and PSF volume was used as a parameter for filtering nonmatching microbubble pairs in this study. Nevertheless, using high volume rates may still be valuable for improving the SNR and for velocity measurements. In an *in vivo* setup, the concentration and velocity of microbubbles may vary between small and large vessels, while tissue attenuation may significantly reduce the microbubble disruption ratio. Hence, for *in vivo* applications, using a high PRF will create an opportunity to improve the SNR by an increasing number of compounding angles or temporal averaging while maintaining a reasonable frame rate. In the future, the relationship between PRF, microbubble flow velocity, imaging pressure, and compounding strategies should be investigated for different applications and physiological flow rates.

## V Conclusion

The main limitation of localization-based SR-US imaging performed in 2-D is the lack of super-resolution in the elevation direction. In this study, this issue was addressed by using a bespoke 2-D sparse array that achieved an estimated localization precision of 18 *μ*m in the worst imaging plane, which is approximately 22 times smaller than the wavelength. Compounded plane-wave imaging with a volume rate of 500 Hz enabled super-resolution imaging in all spatial directions with an image acquisition time of 12 s. The structure of two 200-*μ*m, smaller than half wavelength, tubes arranged in a double helix shape was super-resolved, and flow velocities within these tubes were estimated. The 3-D subdiffraction imaging was achieved *in vitro* using the 2-D sparse array probe.

## Supplementary Material

Video S1

Video S2

Video S3

Video S4
